# SEOM clinical guidelines for the treatment of non-small cell lung cancer (2018)

**DOI:** 10.1007/s12094-018-1978-1

**Published:** 2018-11-17

**Authors:** M. Majem, O. Juan, A. Insa, N. Reguart, J. M. Trigo, E. Carcereny, R. García-Campelo, Y. García, M. Guirado, M. Provencio

**Affiliations:** 1 Medical Oncology Department, Hospital de la Santa Creu i Sant Pau, Sant Antoni Maria Claret 167, 08025 Barcelona, Spain; 2 Medical Oncology Department, Hospital Universitari i Politècnic La Fe, Valencia, Spain; 3 Medical Oncology Department, Hospital Clínico Universitario de Valencia, Valencia, Spain; 4 Medical Oncology Department, Hospital Clínic, IDIBAPS, Universitat de Barcelona, Barcelona, Spain; 5 Medical Oncology Department, Hospital Universitario Virgen de la Victoria, Málaga, Spain; 6 Medical Oncology Department, Institut Català d’Oncologia Badalona-Hospital Germans Trias i Pujol, Badalona, Spain; 7 Medical Oncology Department, Complexo Hospitalario Universitario A Coruña, Coruña, Spain; 8 Medical Oncology Department, Institut d’Investigació i Innovació Parc Taulí I3PT, Universitat Autònoma de Barcelona, Parc Taulí Hospital Universitari, Sabadell, Spain; 9 Medical Oncology Department, Hospital General Universitario de Elche, Elche, Spain; 10 Medical Oncology Department, Hospital Universitario Puerta de Hierro-Majadahonda, Universidad Autónoma de Madrid, Madrid, Spain

**Keywords:** NSCLC, Chemotherapy, Immunotherapy, Targeted therapies, Radiotherapy

## Abstract

Non-small cell lung cancer (NSCLC) accounts for up to 85% of all lung cancers. The last few years have seen the development of a new staging system, diagnostic procedures such as liquid biopsy, treatments like immunotherapy, as well as deeper molecular knowledge; so, more options can be offered to patients with driver mutations. Groups with specific treatments account for around 25% and demonstrate significant increases in overall survival, and in some subgroups, it is important to evaluate each treatment alternative in accordance with scientific evidence, and even more so with immunotherapy. New treatments similarly mean that we must reconsider what should be done in oligometastatic disease where local treatment attains greater value.

## Methodology

Relevant studies published in peer review journals were used for the guideline elaboration. The Infectious Diseases Society of America grading system was used to assign levels of evidence and grades of recommendation.

## Diagnosis

### Diagnosis: pathology and molecular testing

The pathological diagnosis of non-small cell lung cancer (NSCLC) should be made according to the World Health Organization (WHO) classification [1]. The International Association for the Study of Lung Cancer (IASLC) provided adenocarcinoma classification as well as key recommendations for the management of small biopsies and cytology [2]. For therapeutic implications, specific subtyping of NSCLC is strongly recommended whenever possible. Limited diagnostic workup is also recommended to preserve as much tissue as possible for further molecular assessments.

The Spanish Society of Medical Oncology and the Spanish Society of Pathology published evidence-based recommendations for molecular testing in lung cancer [3]. Genetic profiling of NSCLC advanced disease is recommended in daily clinical practice by both ESMO [4] and ASCO [5] guidelines, as it has demonstrated to have an impact on patients’ outcomes (I,A). New molecular guidelines recommend to include upfront ROS-1 testing along with EGFR and ALK in stage IV non-SCC and endorse to include other additional genes such as BRAF, MET, HER2, KRAS and RET for laboratories that perform next-generation sequencing (NGS) testing [5]. Immunohistochemistry can be considered as an alternative to fluorescence in situ hybridization for ALK and/or ROS1 testing.

In EGFR mutant patients progressing on first- or second-generation EGFR TKI, the detection of EGFR T790M secondary resistance mutations in tumor tissue is recommended (I,A). Liquid biopsies or molecular DNA profiling in blood (ctDNA) is currently accepted as a good surrogate for EGFR testing in tissue (II,A), enabling clinicians to collect samples in a non-invasive approach [6, 7].

All patients with advanced NSCLC should, at baseline, have their tissue assessed for programmed cell death 1 ligand (PD-L1) expression by IHC test for selecting patients for anti-programmed death 1 (PD-1) or anti-PD-L1 treatment [8].

### Disease staging

In NSCLC, the following staging work-up is highly recommended:

- Clinical history, including smoking and family history; physical examination, performance status (PS) and weight loss should be assessed.
- Blood test, including hematology, renal and hepatic function.
- Bronchoscopy.
- Chest and upper abdomen (including liver and adrenal glands) computerized tomography (CT).
- Brain CT or magnetic resonance imaging (MRI) is recommended for patients undergoing radical treatment, in patients with EGFR mutation or ALK translocation or if there are neurological symptoms in the physical examination.
- Bone scan is recommended if there is bone pain, high serum calcium or high alkaline phosphatase.
  In patients undergoing potentially radical treatment, additional recommendations should be considered:
- Whole-body FDG-positron emission tomography (PET–CT).
- Pulmonary function tests.
- Ergospirometry if the pulmonary function tests are not normal.
- Chest MRI in Pancoast tumour.
- Invasive mediastinal staging, endobronchial ultrasound-guided fine-needle aspiration and/or endoscopic ultrasound-guided fine-needle aspiration are recommended in patients with suspected mediastinal or hilar lymph nodes (LNs) in the PET–CT. For patients with suspected LNs on PET–CT and negative EBUS/EUS results, an additional mediastinoscopy is recommended. In patients with no suspected LN on the PET-CT, invasive mediastinal staging is also recommended in patients with enlarged mediastinal LNs (≥ 1.5 cm), in tumors ≥ 3 cm and/or in patients with central tumors.
- Histological and cytological confirmation is strongly recommended in the presence of pleural/pericardial effusion or isolated metastatic site.

## Staging system

During the 16th World Congress of Lung Cancer, the International Association for the Study of Lung Cancer (IASLC) proposed the TNM 8th edition that was accepted by the Union for International Cancer Control (UICC) and the American Joint Committee on Cancer (AJCC) [9]. The TNM 8th edition is effective since January 2017 (Table 1). The most striking changes in the TNM 8th edition are the further subdividing and detailing of both T and M stages, although the consequences for therapeutic approach are not yet obvious in all situations.

**Table 1:** TNM classification 8th edition

Stage	T	**N**	M
Occult	TX	N0	M0
0	Tis	N0	M0
IA1	T1a(mi)/T1a	N0	M0
IA2	T1b	N0	M0
IA3	T1c	N0	M0
IB	T2a	N0	M0
IIA	T2b	N0	M0
IIB	T1a-T2b	N1	M0
	T3	N0	M0
IIIA	T1a-T2b	N2	M0
	T3	N1	M0
	T4	N0/N1	M0
IIIB	T1a-T2b	N3	M0
	T3/T4	N2	M0
IIIC	T3/T4	N3	M0
IVA	Any T	Any N	M1a/M1b
IVB	Any T	Any N	M1c

## Stage I–II

A multidisciplinary tumor board evaluation of NSCLC patients with stage I-II disease is strongly recommended, even non-surgical patients. It has to include a preoperative cardiopulmonary assessment.

### Surgery

For stage I–II NSCLC patients and no medical contraindications to surgery, surgical resection remains the treatment of choice, yielding the best potential choice of cure for these patients (IB).

The type of surgery resection depends on the extension of the disease, the location of the tumor and the preoperative evaluation:

- In stage I–II medically fit NSCLC patients, lobectomy or anatomic pulmonary resection is recommended rather than sublobar resection (I,B). Systematic mediastinal lymph node dissection is recommended over selective sampling lymph node dissection for accurate pathologic staging [10] (IB). For stage II patients undergoing anatomic resection, mediastinal lymph node dissection may provide additional survival benefit over sampling [11] (II,B).
- A sublobar resection (anatomical segmentectomy) is recommended over nonsurgical therapy for patients who cannot tolerate a lobar resection due to decreased pulmonary function or comorbid disease (I,B).
- For patients with a stage I predominantly ground glass opacity with lesion ≤ 1 cm, sublobar resection with negative margins is suggested over lobectomy (I,B).
- Reresection is recommended for patients with positive margins in resected stage I–II NSCLC patients. If it is not possible, postoperative radiotherapy may be considered [12].

### Adjuvant therapy

Overall survival (OS) benefit of adjuvant treatment is limited to cisplatin-based chemotherapy in completely resected fit stage II–III patients [13].

- Four cycles of cisplatin-based chemotherapy following complete resection in stage II NSCLC patients remain the standard of care in adjuvant setting, offering a 5% OS benefit [13] (I,A).
- Stage I (7th TNM edition) NSCLC patients do not benefit from adjuvant therapy except those patients with tumors > 4 cm [5, 14] (I,C).
- In elderly fit patients, adjuvant platinum-based chemotherapy should be considered.
- Postoperative radiotherapy (PORT) is not indicated in completely resected stage I–II NSCLC patients [15] (I,A-II,A).

### Neoadjuvant therapy

Preoperative chemotherapy has the potential role to reduce tumor size, increase operability, and eliminate micrometastases. A meta-analysis with 15 randomized trials showed a significant benefit of preoperative chemotherapy on OS (representing an absolute survival improvement of 5% at 5 years [16]. Although neoadjuvant chemotherapy has similar impact on OS than adjuvant chemotherapy, more conclusive evidence favors adjuvant treatment (I,B).

### Stereotactic ablative radiotherapy (SART)

SART is recommended for medically inoperable NSCLC patients with node negative tumors ≤5 cm (2C). Several non-randomized studies suggest that SART might be a suitable option for operable patients older than 75 years [17] (II,C).

### Other adjuvant treatments

Adjuvant EGFR TKI in patients with EGFR mutation has not demonstrated a survival benefit yet. Several trials in patients with EGFR mutations or ALK translocations in adjuvant setting are ongoing [18].

## Stage III

**Fig. 1:**
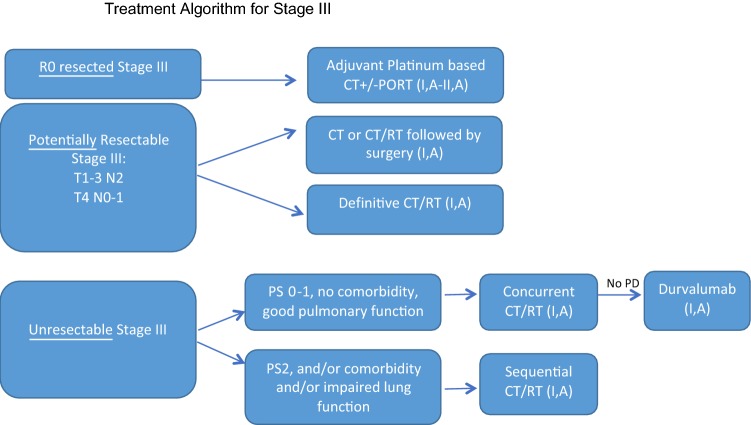
Treatment algorithm for Stage III

Stage III NSCLC is a heterogeneous and complex disease that has been classified into different subgroups: resectable, potentially resectable and unresectable locally advanced NSCLC. Treatment decision should be taken by an experienced multidisciplinary team (Fig. 1).

### Resectable and potentially resectable NSCLC

- In patients with R0 resected stage III NSCLC, 4 cycles of adjuvant platinum-based chemotherapy should be given (preferably cisplatin doublet) [19] (I,A).
- In patients with pathological N2 NSCLC, PORT appears to improve OS in non-randomized analysis, and it is usually administered after adjuvant chemotherapy (II,A).
- In patients with potentially resectable disease, the optimal treatment strategy remains unclear. Several phase III trials and a meta-analysis showed that induction therapy followed by surgery might be better than surgery alone [20]. Surgery has been compared to radiotherapy in patients with tumor response after induction chemotherapy, without differences in overall survival [21]. Surgery was also compared to radiotherapy after induction chemoradiotherapy in the Lung Intergroup Trial 0139 showing better progression-free survival in the surgery arm, with no differences in OS except in the unplanned analysis in the subset of patients who underwent lobectomy [22]. The optimal chemotherapy regimen has not been established in randomized trials, although cisplatin-based chemotherapy is recommended.
- In case of superior sulcus (Pancoast) tumors, concurrent chemoradiation followed by surgery is the preferred option [23] (Table 2).

**Table 2:** Summary of recommendations

Diagnosis	WHO classification for pathological diagnosis is required and also IASLC classification of adenocarcinoma For therapeutic implications, specific subtyping of NSCLC is strongly recommended Molecular testing in stage IV non-SCC should include EGFR mutations, ALK and ROS-1 translocations by a validated technique In patients progressing to first or second generation EGFR TKI determination of EGFR T790M in plasma or tissue should be performed PD-L1 expression should be test to all patients with advanced NSCLC at baseline
Staging	Comprehensive evaluation must include thorax and upper abdomen CT More extensive evaluations are recommended if a radical approach is considered Stage disease must be classified using the TNM 8th edition
Stage I–II	Patients should be evaluated in a multidisciplinary tumor board
Medically fit for surgery	Lobectomy or anatomic pulmonary resection plus systematic mediastinal lymph node dissection
Medically inoperable, node negative NSCLC tumours ≤ 5 cm	SART
Adjuvant chemotherapy (four cycles of cisplatin-based chemotherapy)	Recommended in stage II Not recommended in stage I 7th TNM edition (except T > 4 cm)
Post operative radiotherapy (PORT)	Not indicated in completely resected stage I–II
Stage III	Treatment decision should be taken by an experienced multidisciplinary team
Completely resected	Adjuvant chemotherapy (four cycles of adjuvant cisplatin-based chemotherapy) ± PORT
Potentially resectable	Resection followed by adjuvant chemotherapy Induction chemotherapy or chemoradiotherapy followed by surgery
Unresectable stage III	Medically fit: concurrent chemoradiotherapy with cisplatin-based chemotherapy Sequential chemoradiotherapy if concurrent treatment is not feasible PCI is not indicated Durvalumab if no progressive disease after concurrent chemoradiotherapy
Stage IV	
Stage IV without driver mutations	
Fist line	
PD-L1 ≥ 50%	Pembrolizumab Note: Combination of immunotherapy plus standard chemotherapy may be considered
PD-L1 < 50% or unknown	Platinum-based chemotherapy based on tumor histology:
Squamous cell carcinoma (SCC)	Platinum-based doublets (4, up to 6 cycles) Immunotherapy (atezolizumab or pembrolizumab) and carboplatin plus paclitaxel or nab-placlitaxel)^(#)^
Non-squamous-cell carcinoma (non-SCC)	Platinum-based doublet: Cisplatin/pemetrexed has more efficacy and less toxicity than cisplatin/gemcitabine Bevacizumab added to a platinum doublet. if there are no contraindications Pemetrexed maintenance Immunotherapy (atezolizumab^(#)^ or pembrolizumab) plus standard chemotherapy
Elderly	Comprehensive geriatric assessment is highly recommended
Fit patients	Decision according to histology and PD-L1 expression levels
Unfit or comorbidities	Single agent chemotherapy
PS 2	Combination therapy Single-agent therapy Best supportive care
PS 3–4	Best supportive care
Second line	
PS 0–2	
If no prior immunotherapy	Pembrolizumab (PD-L1 ≥ 1%), nivolumab or atezolizumab
If prior immunotherapy	Platinum doublets
If contraindication for immunotherapy	Docetaxel–nintedanib (non-SCC) Docetaxel (SCC, non-SCC) Pemetrexed (non-SCC)
If prior immunotherapy alone	Platinum doublets
If prior immunotherapy + CT	Docetaxel–nintedanib (non-SCC) Docetaxel (SCC, non-SCC) Pemetrexed (non-SCC)
PS 3–4	Best supportive care
Stage IV with driver mutations	
EGFR mutation	
First-line EGFR TKI	Erlotinib, gefitinib, afatinib, dacomitinib^(#)^, osimertinib
Brain metastasis	Osimertinib
After EGFR TKI progression	
Clinical benefit maintained or oligoprogressive disease	Continuation with the EGFR TKI
T790 M positive	Osimertinib (if not previously given)
T790 M negative	Platinum-based chemotherapy
ALK mutation	
First-line ALK TKI	Alectinib, brigatinib^(#)^, crizotinib or ceritinib
Progression to crizotinib	Ceritinib, alectinib or brigatinib^(#)^
Brain metastasis	Alectinib, brigatinib^(#)^ or lorlatinib^(#)^
Other genetic alterations	
ROS-1	Crizotinib
B-RAF^v600^	Dabrafenib plus trametinib
Oligometastatic disease	
	Systemic therapy and local ablative strategies Local ablative strategies and TKI-continuation if clinical benefit is still retained (if actionable mutation)
Follow-up	
	Smoking cessation counseling
Curative intent	
Surgery	Medical history, physical examination and spiral chest CT scan every 6–12 months for 2 years and annually thereafter
SART	Medical history, physical examination and spiral chest CT scan every 6 months for 3 years and annually thereafter PET–CT ± biopsy if recurrence is suspected
Advanced disease	
	Early palliative care Evaluation of response every 6–12 weeks

(#) Not EMA approved

### Unresectable NSCLC

Unresectable LA-NSCLC includes stage IIIA N2 (bulky and/or multiple nodal involvement), stage IIIB and IIIC.

- Concurrent chemoradiotherapy is the treatment of choice for medically fit patients (I,A). Several randomized clinical trials and a meta-analysis have shown a higher 5-year survival rates favoring this strategy over sequential approaches [24].
- Cisplatin-based combinations are recommended for medically fit patients (usually with etoposide or vinorelbine) [24].
- Radiotherapy is usually given at a dose of 60–66 Gy in 30–33 fractions over 6–7 weeks. Higher doses are not recommended outside of clinical trials [25].
- If concurrent chemoradiotherapy is not feasible due to poor performance status, comorbidities, and/or unfit patients, a sequential approach is a reasonable option [26].
- There is no role for prophylactic cranial irradiation in stage III (II,A).
- In patients with no progressive disease after concurrent chemoradiotherapy, consolidation treatment with Durvalumab for 1 year has shown to improve progression-free survival (PFS) and OS (I,A) [27–29]. The European Medical Agency has recently approved consolidation with Durvalumab in patients with PD-L1 expression ≥ 1% based on an unplanned post hoc analysis.

## Stage IV

### Stage IV without driver mutations (Fig. 2)

#### First-line therapy

- For stage IV, PS 0–1 NSCLC patients without driver mutations whose tumors express PD-L1 at levels of 50% or greater (tumor proportion score (TPS) ≥ 50%), pembrolizumab is recommended in the absence of contraindications to use immunotherapy [30] (I,A).
- For patients with low (TPS < 50%) or unknown PD-L1 expression, chemotherapy with platinum doublets should be considered in all stage IV PS 0–1 NSCLCs without driver mutations (I,A). Data have shown that platinum combination therapy increases OS and improves quality of life (QoL) compared to supportive care, single-agent cisplatin or other monotherapy [31–34].
- Meta-analyses have shown higher response rates (RRs) and a slightly longer OS for cisplatin combinations than for carboplatin combinations [35] (I,B). Carboplatin can be recommended if any contraindication for cisplatin exists.
- Non-platinum regimens have reported lower efficacy than platinum regimens [36] (I,A).
- Recently, results from several phase III trials have shown a significant benefit in terms of efficacy for the addition of immunotherapy to platinum-based chemotherapy regardless of the PD-L1 status [37–41] (I,A-I,B).
- Cisplatin-based combinations and some modalities of treatment will be selected based on tumor histology:

**Fig. 2:**
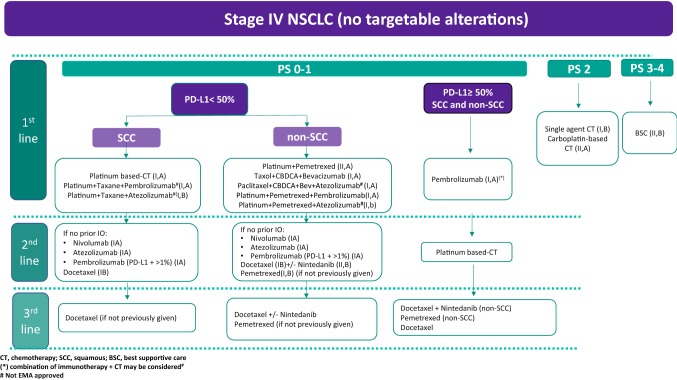
Treatment algorithm for Stage IV with no targetable alterations

##### For squamous cell lung cancer (SCC)

- For PS 0–1 SCC patients, without major comorbidities and with low (TPS < 50%) or unknown PD-L1, platinum-based doublets with the addition of a third-generation cytotoxic agent (gemcitabine, vinorelbine, taxanes) are recommended (I,A). The different combinations have shown comparable efficacy [42].
- Four cycles are recommended, up to a maximum of six cycles in selected cases [43, 44] (I,A).
- The expected toxicity profile should contribute to the selection of the chemotherapy regimen. The nab-paclitaxel/carboplatin regimen has shown in a phase III trial to have higher RRs (with a larger impact in SCC) than paclitaxel/carboplatin and less neurotoxicity (I,B) [45].
- Recently, two randomized phase III trials have shown that the addition of immunotherapy (atezolizumab or pembrolizumab) to standard first-line chemotherapy (carboplatin plus paclitaxel or nab-placlitaxel) in SCC, results in significantly longer PFS with atezolizumab (I,B) [38] and OS and PFS with pembrolizumab (I,A) [40] than chemotherapy alone, regardless of PD-L1 expression. It is important to underline that these combinations were not approved by the European Medical Agency when this guideline was submitted.

##### For non-squamous cell lung cancer (Non-SCC)

- Any platinum-based doublets with a third-generation agent can be used in non-SCC patients with low (TPS < 50%) or unknown PD-L1 [42] (I,A).
- Pemetrexed-based combination chemotherapy represents a therapeutic option. This regimen showed a slight but significant survival benefit compared with gemcitabine or docetaxel-based combinations (results coming from a meta-analysis and a preplanned subgroup analysis of a randomized phase III trial) [46, 47] (II,A).
- Bevacizumab/paclitaxel/carboplatin combination chemotherapy followed by maintenance bevacizumab has shown improvement in OS in two randomized clinical trials and, therefore, it can be offered to patients with advanced PS 0–1 non-SCC and no contraindications for antiangiogenic treatment [6, 48] (I,A).
- Maintenance therapy can be considered in those PS 0–1 patients with at least stable disease and who have recovered from residual toxicity after first-line chemotherapy:
  - Pemetrexed switch maintenance could be considered after four cycles of non-pemetrexed platinum-based chemotherapy [49] (I,B).
  - Pemetrexed continuation maintenance should be considered in patients having disease control after four courses of pemetrexed platinum-based chemotherapy [50] (I,A).
- Recently, three randomized phase III trials have shown that the addition of immunotherapy (pembrolizumab or atezolizumab) to standard first-line chemotherapy (pemetrexed platinum-based combination or bevacizumab plus chemotherapy) in non-SCC resulted in significantly longer OS ± PFS than chemotherapy alone, regardless of PD-L1 expression [37, 39, 41]. It is important to underline that pembrolizumab with pemetrexed and platinum-based chemotherapy was the only combination approved by the European Medical Agency when this guideline was submitted.

### Second-line therapy

Patients clinically or radiologically progressing after first-line therapy, with a PS 0–1 and appropriate PS 2, should be offered second-line treatment (I,A). Second-line treatment should be individualized and treatment duration should be subject to tolerability and clinical benefit.

- In patients with metastatic non-SCC and SCC who have not received prior immunotherapy, and with no contraindications, single-agent pembrolizumab (PD-L1 TPS ≥ 1%), nivolumab or atezolizumab is recommended (I,A). This recommendation is based on data from the main Phase III trials, showing significant improvements in OS and tolerability of immunotherapy agents when compared to single-agent docetaxel [51–54].
- Nintedanib added to docetaxel has demonstrated a significant OS benefit as compared with docetaxel alone in previously treated stage IV, PS 0–1 adenocarcinoma, particularly in those patients progressing within 9 months after start of first-line therapy [55] (II,B).
- Docetaxel, or pemetrexed have demonstrated improvement in terms of OS and QoL (I,B) and are recommended for those patients with contraindications to immunotherapy or nintedanib combination therapy (non-SCC) [56, 57].
- In patients who have received an immune checkpoint inhibitor as first-line therapy, platinum doublets are recommended (I,B).
- For those patients who have received first-line conventional chemotherapy and immune therapy, single agent, docetaxel, pemetrexed (non-SCC) or docetaxel plus nintedanib (non-SCC) could be considered (IIB).
- There is no sufficient evidence to recommend the use of cytotoxic drugs as fourth-line therapy or beyond; patients should be considered to be included in clinical trials, and continued best supportive care.

### Elderly and PS2

Age should not be considered as a decisive factor for treatment selection, and Comprehensive Geriatric Assessment would help to ascertain the true biological status [58].

- For those elderly fit patients with PS 0–1 and adequate organ function, first-line treatment decision should be based according to histology and PD-L1 expression levels [59] (I,B). Single agent chemotherapy (vinorelbine, gemcitabine, docetaxel) is recommended for those with comorbidities or unfit patients [60] (IB).
- For patients with PS 2, chemotherapy prolongs OS compared to best supportive care (BSC) [61] (I,B). In an individualized-based decision, combination therapy, single-agent therapy, or palliative therapy alone may be used for PS 2 patients. In the first-line setting, platinum-based doublets (preferably carboplatin) have superior efficacy to monotherapy, despite higher toxicity rates [62, 63] (II,A).
- Unfit patients with PS 3–4 should not receive active treatment regardless of age because no benefit has been demonstrated. Supportive care is recommended (II,B).

## Stage IV with driver mutations (Fig. 3)

### EGFR mutation

#### First-line setting

- EGFR TKIs (gefitinib, erlotinib, afatinib) have shown superior PFS, RR, toxicity profile and QoL for EGFR TKIs as first-line treatment compared with platinum-based doublets (I,A) [64, 65]. Only a prespecified subanalysis showed a significant improvement in OS favoring afatinib in patients with Del19 mutations [65].
- Patients with PS 3–4 may also be offered an EGFR TKI, as they are likely to receive a similar clinical benefit to patients with good PS (III,A).
- Results from direct comparison of first-, second- and third-generation EGFR TKIs in previously untreated patients have been reported. Although a benefit in terms of PFS has been demonstrated for third-generation TKIs osimertinib (I,A) and dacomitinib (I,A) [66–68], to date only dacomitinib has shown a significant OS advantage (I,A) [69]. However, grade 3–4 treatment-related adverse events were significantly higher with dacomitinib. OS data from the FLAURA trial comparing osimertinib versus standard of care are still immature [67].
- An exploratory data on brain disease suggest that the probability of experiencing a progression on central nervous system (CNS) was lower with osimertinib and provided a higher intracranial activity (II,B) [70].
- Combinations of bevacizumab and erlotinib were also explored in the first-line setting demonstrating a significant increase in PFS but only a slight trend of OS improvement with the combination [71–73] (I,B).
- Combination of pemetrexed-carboplatin and gefitinib has demonstrated a significant increase in PFS and OS in japanese population [74] (I,B).

**Fig. 3:**
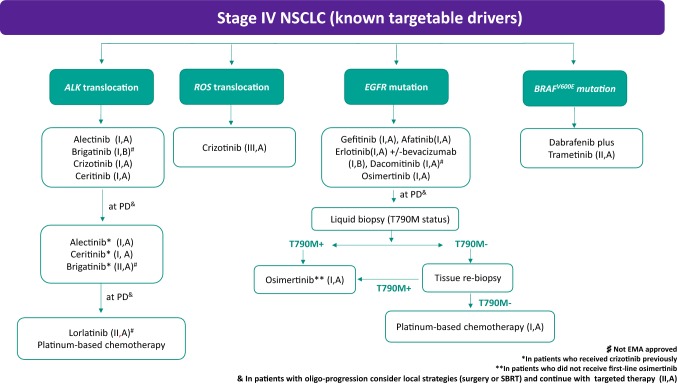
Treatment algorithm for Stage IV with known targetable drivers

### After EGFR TKI progression

- Patients might benefit of continuation with the EGFR TKI, especially if clinical benefit is maintained from a sustained EGFR oncogenic blockade [7] or if there is an oligoprogressive disease treatable with local strategies (SART or surgery) (II,A) [75].
- EGFR Exon 20, T790 M mutation, is the main mechanism of acquired resistance after first- or second-generation EGFR TKIs [76]. Osimertinib has demonstrated greater efficacy over platinum-based chemotherapy (I,A) [77].
- For patients with systemic symptomatic progression in whom T790 M cannot be detected or who have progressed to osimertinib, platinum-based chemotherapy remains the standard of care (II,A). The combination of atezolizumab plus bevacizumab plus chemotherapy has demonstrated a significant PFS benefit in the subgroup of patients with EGFR mutation (III,A) [50].
- Continuation of EGFR TKI with platinum-based chemotherapy does not impact on PFS nor on OS [78] (I,A).

### ALK translocation

#### First-line setting

- First-line treatment with ALK TKIs is the preferred treatment (I,A). Crizotinib and ceritinib have shown a significant statistical improvement in terms of PFS and RR compared with chemotherapy in randomized phase III trials (I,A) [79, 80].
- Alectinib (I,A) and brigatinib (I,B) have shown a significant improvement in PFS versus crizotinib and, therefore, are the preferred first-line options. Grade 3–5 adverse events were higher for patients treated with crizotinib [81, 82]. It is important to underline that brigatinib was not approved by the European Medical Agency when this guideline was submitted.
- Chemotherapy is indicated (III,B) in patients whose ALK results are not available and urgent systemic treatment is required. Treatment plan should be reassured when genotypic results were available.
- For patients who received chemotherapy in the first-line, crizotinib should be recommended as second-line treatment (I,A) [83]. Alectinib and ceritinib should also be considered, although no specific trials have been conducted.

### After ALK TKI progression

- For patients who develop resistance or are intolerant to crizotinib, ceritinib (IA), alectinib (IA) or brigatinib (IIA) can be recommended. Ceritinib and alectinib have shown a significant improvement in median PFS and less adverse events than chemotherapy. Brigatinib has shown a favorable PFS in a crizotinib-refractory ALK-positive phase II trial [84–86].
- Lorlatinib has shown activity in patients who have progressed on next-generation ALK TKI (ceritinib, alectinib or brigatinib) [87] (II,A).
- Ensartinib and entrectinib have also been demonstrated activity in previously treated patients in early phase trials [88, 89].
- For patients with systemic symptomatic progression to ALK TKI, platinum-based chemotherapy remains the standard of care (II,A). The combination of atezolizumab plus bevacizumab plus chemotherapy has demonstrated a significant PFS benefit (III,B) [50].

### Brain metastases

- Alectinib, brigatinib and lorlatinib have shown greater activity in CNS disease. In the ALEX trial, fewer patients treated with alectinib (12%) had CNS progression than crizotinib (45%). In the ALTA-1 trial, intracranial RR was 78% for brigatinib versus 29% for crizotinib [82].
- For asymptomatic or patients who became asymptomatic with steroids, brain-penetrable ALK TKIs may be used and local treatments may be deferred (I,B).

### *ROS*-*1* and other rare targetable genetic alterations

- Crizotinib is indicated for the treatment of *ROS*-*1*-positive advanced NSCLC based on the results of a single-arm trial in 50 patients [90] (III,A).
- Dabrafenib–Trametinib is indicated for the treatment of advanced NSCLC BRAF^V600E^ mutation based on results from non-comparative studies in pretreated or naïve patients [91, 92] (II,A).
- New investigational drugs have shown activity in early clinical trials targeting oncogenic drivers such as crizotinib, tepotinib or capmatinib (MET amplified, MET^e14^ mutation), LOXO-292 and BLU-667 (RET), entrectinib (NRTK, ROS, ALK fusions), LOXO-101 or larotrectinib (NRTK fusions) [93] and ado-trastuzumab emtansine (HER2 mutations). However, none of these targeted drugs have an official regulatory approval by EMA except the orphan drug designation of LOXO-101 in NTRK fusion tumors.

## Oligometastatic NSCLC

The oligometastatic state consists of patients with metastasis limited in number and location. The number of metastases ranges from a single metastatic lesion to a single organ with multiple metastases or to multiple lesions in multiple organs. The most accepted number of metastatic lesions is up to five and, most important, these should be suitable to radical treatment by local therapy: surgical resection, SART or both. The oligometastatic disease comprises four different settings [94]:

1. Metastatic lesions limited in number and location at diagnosis, all the lesions including the primary tumor are suitable to radical therapy.
2. Multiple metastases that are transformed into an oligometastatic disease after systemic treatment due to response, and all lesions can be managed with radical intent.
3. The primary tumor and most areas of metastatic disease remain controlled, but one or a limited number of metastases progress while systemic therapy.
4. Oligorecurrence occurs in patients treated with curative intent and metachronously present 1–5 metastastic lesions suitable to ablative therapy.
  Patients with oligometastatic disease at diagnosis should be treated with systemic therapy and local consolidative ablative therapy (LAT) to primary and all metastatic sites. Two phase II studies showed that LAT after systemic therapy increased PFS vs no further local treatment [95, 96] (I,A).
  Patients with actionable mutations receiving targeted therapies who progress on isolated site can be treated with LAT [75, 97] (II,A).

## Management and follow-up

- Smoking cessation counseling is encouraged in any stage as it leads to superior treatment outcomes since smoking may impact on drugs’ bioavailability (II,A).
- There is not an established consensus regarding the most optimal follow-up in patients with NSCLC. However, due to the inherent aggressiveness of the disease, a close follow-up is advised.

### Follow up in patients after curative treatment:

- NSCLC patients treated with radical intent must be followed to identify treatment-related complications, detection of treatable relapse or occurrence of second primary lung cancer (III,A).
- In patients with curative surgery, a close follow-up visit including medical history, physical examination and chest CT is recommended every 6–12 months for the first 2 years and annually thereafter (III,B).
- For patients treated with SART with radical intent, CT scans every 6 months for 3 years are recommended and annually thereafter (III,B). PET–CT ± biopsy is endorsed when recurrence is suspected based on chest CT To discriminate from focal fibrosis (III,B).
- Routine surveillance with blood test, FDG-PET imaging or another radiological assessment is not endorsed (II,D).

### Follow up in patients with advanced disease:

- Early palliative care is strongly recommended [98] (I,A).
- Evaluation of response is recommended every 6–12 weeks after therapy initiation, using the same baseline radiographic method. The frequency of the radiologic assessment can be tailored for patients benefiting long time on targeted agents (III,B).
- For patients eligible for successive lines that respond to first-line treatment, it is advisable to undergo clinical and/or radiological evaluation 6 weeks after finishing treatment and then every 6–12 weeks to enable second-line therapy to commence promptly (III,B).
